# RSK2 Is a Modulator of Craniofacial Development

**DOI:** 10.1371/journal.pone.0084343

**Published:** 2014-01-08

**Authors:** Virginie Laugel-Haushalter, Marie Paschaki, Pauline Marangoni, Coralie Pilgram, Arnaud Langer, Thibaut Kuntz, Julie Demassue, Supawich Morkmued, Philippe Choquet, André Constantinesco, Fabien Bornert, Matthieu Schmittbuhl, Solange Pannetier, Laurent Viriot, André Hanauer, Pascal Dollé, Agnès Bloch-Zupan

**Affiliations:** 1 Institute of Genetics and Molecular and Cellular Biology (IGBMC), Centre National de la Recherche Scientifique (UMR 7104), Institut National de la Santé et de la Recherche Médicale (U 964), University of Strasbourg, Illkirch, France; 2 Team «Evo-Devo of Vertebrate Dentition», Institut de Génomique Fonctionnelle de Lyon, Unité Mixte de Recherche 5242 Centre National de la Recherche Scientifique, Ecole Normale Supérieure de Lyon, Claude Bernard Lyon 1 University, Lyon, France; 3 Faculty of Dentistry, University of Strasbourg, Strasbourg France; 4 UF6237 Preclinical Imaging Lab, Biophysics and Nuclear Medicine, Hôpitaux Universitaires de Strasbourg (HUS), Strasbourg, France; ICube, CNRS, University of Strasbourg, Strasbourg, France; 5 Reference Centre for Orodental Manifestations of Rare Diseases, Pôle de Médecine et Chirurgie Bucco-dentaires, Hôpitaux Universitaires de Strasbourg (HUS), Strasbourg, France; 6 INSERM U1121, "Biomaterials and Bioengineering", University of Strasbourg, Strasbourg, France; 7 Faculty of Dentistry, Khon Kaen University, Khon Kaen, Thailand; Deakin School of Medicine, Australia

## Abstract

**Background:**

The *RSK2* gene is responsible for Coffin-Lowry syndrome, an X-linked dominant genetic disorder causing mental retardation, skeletal growth delays, with craniofacial and digital abnormalities typically associated with this syndrome. Craniofacial and dental anomalies encountered in this rare disease have been poorly characterized.

**Methodology/Principal Findings:**

We examined, using X-Ray microtomographic analysis, the variable craniofacial dysmorphism and dental anomalies present in *Rsk2* knockout mice, a model of Coffin-Lowry syndrome, as well as in triple *Rsk1,2,3* knockout mutants. We report *Rsk* mutation produces surpernumerary teeth midline/mesial to the first molar. This highly penetrant phenotype recapitulates more ancestral tooth structures lost with evolution. Most likely this leads to a reduction of the maxillary diastema. Abnormalities of molar shape were generally restricted to the mesial part of both upper and lower first molars (M1). Expression analysis of the four *Rsk* genes (*Rsk1*, *2*, *3* and *4*) was performed at various stages of odontogenesis in wild-type (WT) mice. *Rsk2* is expressed in the mesenchymal, neural crest-derived compartment, correlating with proliferative areas of the developing teeth. This is consistent with RSK2 functioning in cell cycle control and growth regulation, functions potentially responsible for severe dental phenotypes. To uncover molecular pathways involved in the etiology of these defects, we performed a comparative transcriptomic (DNA microarray) analysis of mandibular wild-type versus *Rsk2-/Y* molars. We further demonstrated a misregulation of several critical genes, using a *Rsk2* shRNA knock-down strategy in molar tooth germs cultured *in vitro*.

**Conclusions:**

This study reveals RSK2 regulates craniofacial development including tooth development and patterning via novel transcriptional targets.

## Introduction

**Figure 1:**
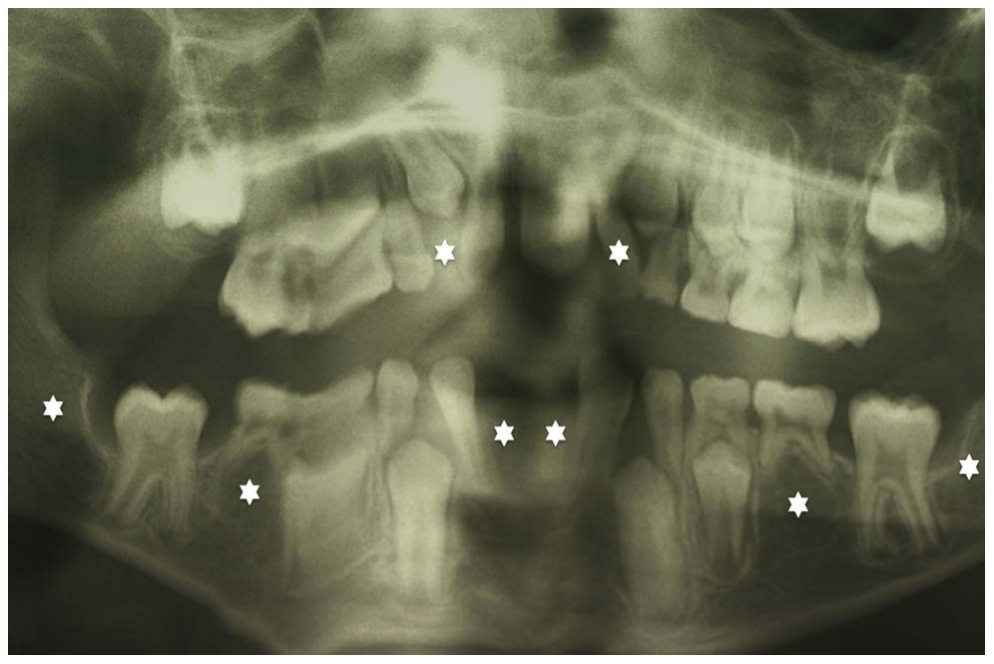
Dental anomalies encountered in Coffin-Lowry syndrome. This panoramic radiograph shows oligodontia (more than six permanent missing teeth (white stars: 12, 22, 41, 31, 45, 35, 47, 37). The deformations observed on this panoramic radiograph are linked to movements during the X-Ray acquisition process and difficulties for handicapped patient to remain still during the procedure.

The ribosomal S6 family of serine/threonine kinases is composed of 4 highly related members in mammals: RSK1 (human chromosome 3), RSK2 (*RPS6KA3*, Xp22.2-p22.1), RSK3 (chromosome 6) and RSK4 (Xq21), which are 75% homologous and are implicated in several important cellular events including proliferation, differentiation, cellular stress response and apoptosis. Mutations in *RSK2* cause X-linked Coffin-Lowry syndrome (OMIM #303600) characterized by psychomotor and growth retardation, with typical facial and digital abnormalities and progressive skeletal malformations like delayed bone development, spinal kyphosis/scoliosis, and sternum/rib protrusins (pectus carinatum) or depression (excavatum) [1], [2], [3]. Orodental findings include a high narrow palate, a midline lingual furrow, malocclusion, hypodontia, and peg shaped incisors (Fig. 1). Premature loss of the primary dentition was also observed, and wide spaced teeth and large medial incisors were reported [1], [4], [5].

RSKs are Ser/Thr protein kinases that act at the distal end of the Mitogen-Activated Protein Kinase/Extracellular signal-Regulated Kinase (MAPK/ERK) signalling pathway. The various RSK proteins are widely expressed, with many cell types expressing several members. RSKs are directly phosphorylated and activated by ERK1/2 in response to several growth factors [6]. In the cytosol, RSK proteins have been shown to phosphorylate substrates including GSK3, L1CAM, the Ras GEF-Sos, IkB, the p34cdc2-inhibitory kinase Myt1, the translation factors eEF2 and eIF4B, and the pro-apoptotic protein BAD [7], [8]. Moreover, upon activation a fraction of the cytosolic RSKs translocate to the nucleus where they are thought to regulate gene expression through phosphorylation of transcription factors such as CREB1, ERa, Nurr77 and SRF, as well as histones [7]. The contribution of each RSK family member to the *in vivo* activation of most of these substrates is currently not well defined.

RSK orthologues have been identified in mouse, rat, chicken, Xenopus and Drosophila [9]. *Rsk2* knockout mice display spatial learning and memory impairment [10] and develop a progressive osteopenia due to impaired osteoblast function [11]. Lack of phosphorylation of the transcription factor ATF4 is responsible for the skeletal abnormalities in *Rsk2* knockout mice [10]. Murine *Rsk2* is highly expressed during somitogenesis, suggesting skeletal and muscle growth and/or patterning roles, potentially leading to the numerous skeletal abnormalities encountered in the human syndrome [12]. It is interesting to note that *Rsk2* shows specific developmental patterns of expression in the maxillary and the mandibular components of the first branchial arch [12], and that craniofacial and dental anomalies are present in the clinical synopsis of Coffin-Lowry syndrome.

The mouse dentition is a powerful and useful model to study the mechanisms leading to human dental anomalies, despite some intrinsic differences. The mouse has a monophyodont dentition that encompasses only four permanently growing incisors and 12 molars separated by a diastema [13]. Most mutant mouse models for human syndromic and non-syndromic genetic disorders displaying dental defects mimicking human pathologic phenotypes [14], although some discrepancies have been reported [15]. In this study, we describe the craniofacial and orodental phenotype of *Rsk2-/Y* knockout mice, as well as triple *Rsk1,2,3-/-* mutants. By employing several additional approaches (expression analysis by *in situ* hybridization, comparative transcriptomic analysis, shRNA knock-down in tooth germ explants) we establish RSK2 regulates defined processes in odontogenesis and craniofacial growth.

## Results

### *Rsk2-/Y* craniofacial phenotype

**Figure 2:**
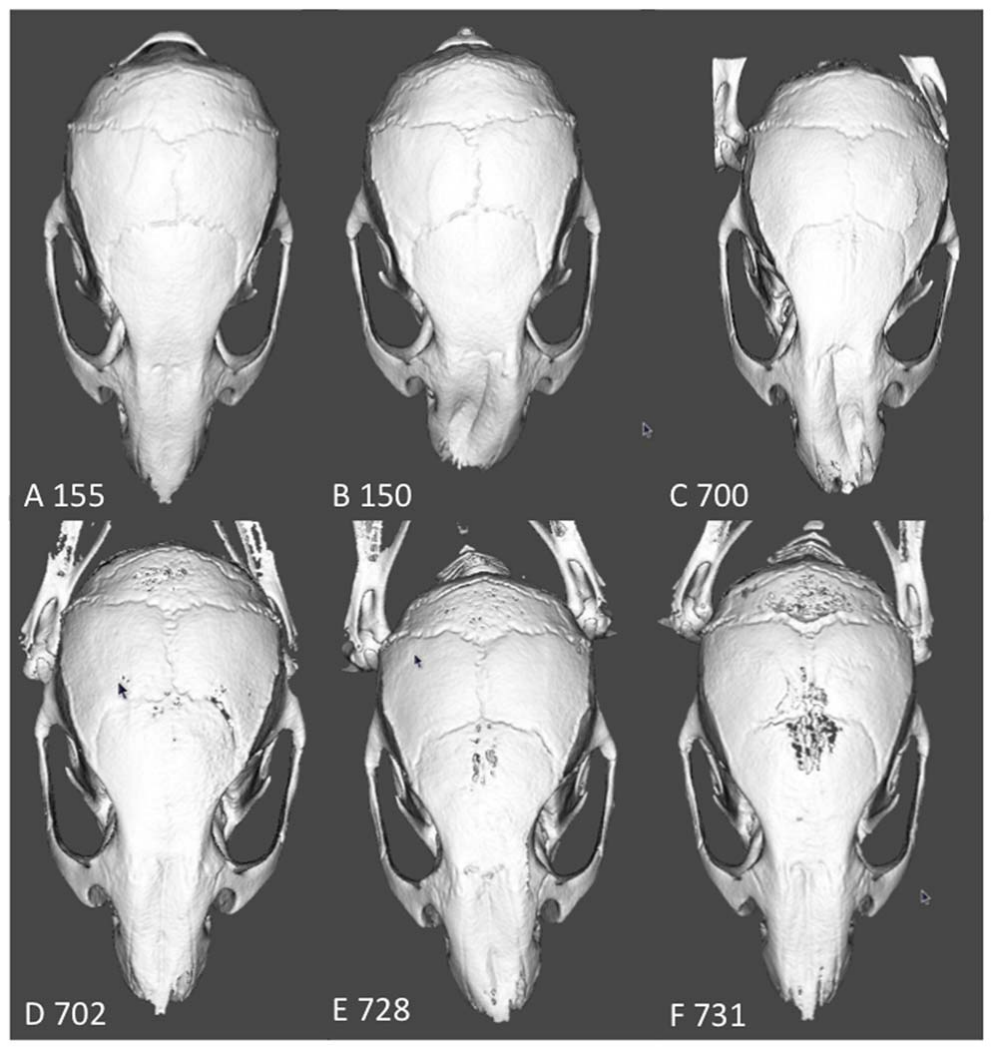
Craniofacial phenotype of *Rsk2-/Y* mice assessed by X-Ray microtomography. The craniofacial phenotype is highly variable, ranging from an almost normal-shaped cranium (mutant 155), although smaller in length, to a very dysmorphic appearance with a lateral nasal deviation. For mutant identification see Table 1.

The craniofacial phenotype was assessed by X-Ray microtomographic imaging and analyzed through euclidean distances measurement and comparison. This phenotype was variable, ranging from normality (mutant mice 702, 731) to obvious craniofacial dysmorphology (mutant mice 150, 700) (Fig. 2). Some mutant mice show an intermediate phenotype, with skulls smaller in length but not in width.

An overall skull length reduction from the nasal to the occipital bone was a collective measure of the sum defects in the nasal, frontal, parietal, interparietal/occipital bones. The frontal bone always exhibited the greatest reductions in length of all skull bones. The interparietal/occipital bones were the least affected (distances 1–4, 1–5, 2–3, 2–4, 2–5 being more reduced than 1–2 or 1–3: see Table 1; measurements were performed according to anatomical landmarks detailed in Fig. S1). A nasal deviation was observed in some mutant mice (Fig. 2B,C,E; Table 1). Craniofacial microtomographic analysis of *Rsk1,2,3-/-* mice was not performed, due to rare availability of these triple mutants. Analysis thus focused on *Rsk2-/Y* mice, a defined genetic model of human Coffin-Lowry syndrome.

**Table 1:** Distances between skull landmarks on wild-type and *Rsk2-/Y* mutant mice.

Distances	Mean WT	STD WT	Mean *Rsk2* mutants	STD *Rsk2* mutants	p-value	
**Between 1 and 2**	**7.10**	**0.59**	**6.84**	**0.35**	**0.03**	
**Between 1 and 3**	**15.05**	**0.46**	**13.92**	**0.59**	**0.01**	
**Between 1 and 4**	**18.59**	**0.41**	**17.24**	**0.57**	**0.005**	
**Between 1 and 5**	**21.87**	**0.49**	**20.48**	**0.48**	**0.005**	
**Between 2 and 3**	**8.07**	**0.45**	**7.25**	**0.47**	**0.005**	
**Between 2 and 4**	**11.72**	**0.38**	**10.69**	**0.45**	**0.005**	
**Between 2 and 5**	**15.21**	**0.35**	**14.13**	**0.31**	**0.005**	
Between L and R 6	12.08	0.17	12.16	0.13	0.47	
Between L and R 7	6.42	0.21	6.45	0.27	0.68	
Between L and R 8	4.77	0.11	4.79	0.73	0.22	
Between L and R 9	6.27	0.25	6.13	0.12	0.06	
Between L and R 10	0.74	0.06	0.71	0.08	0.47	
Between L and R 11	1.05	0.05	1.13	0.11	0.29	
Between L and R 12	10.08	0.37	10.22	0.28	0.29	
Between L and R 15	4.53	0.19	4.72	0.51	0.37	
Between L and R 16	2.29	0.22	2.23	0.23	0.57	
**Between 1 and 6R**	**15.24**	**0.34**	**14.39**	**0.52**	**0.008**	
**Between 1 and 6L**	**15.20**	**0.32**	**14.47**	**0.31**	**0.008**	
**Between 1 and 7R**	**7.92**	**0.28**	**7.18**	**0.58**	**0.008**	
**Between 1 and 7L**	**7.89**	**0.22**	**7.24**	**0.34**	**0.005**	
Between 1 and 8R	6.90	0.24	6.29	0.46	0.88	
Between 1 and 8L	6.90	0.25	6.35	0.29	0.68	
**Between 1 and 9R**	**20.65**	**0.47**	**19.55**	**0.46**	**0.008**	
**Between 1 and 9L**	**20.62**	**0.48**	**19.56**	**0.32**	**0.008**	

In bold are distances significantly different between wild- type (WT) and mutants (all these distances were shorter in mutants). Anatomical landmarks are explained in Fig. S1 (supplementary information). All distances are in millimeters. STD: standard deviation; L: left; R: right.

### *Rsk2-/Y* and *Rsk1,2,3-/-* dental phenotype

#### Phenotype of molar tooth rows

**Figure 3:**
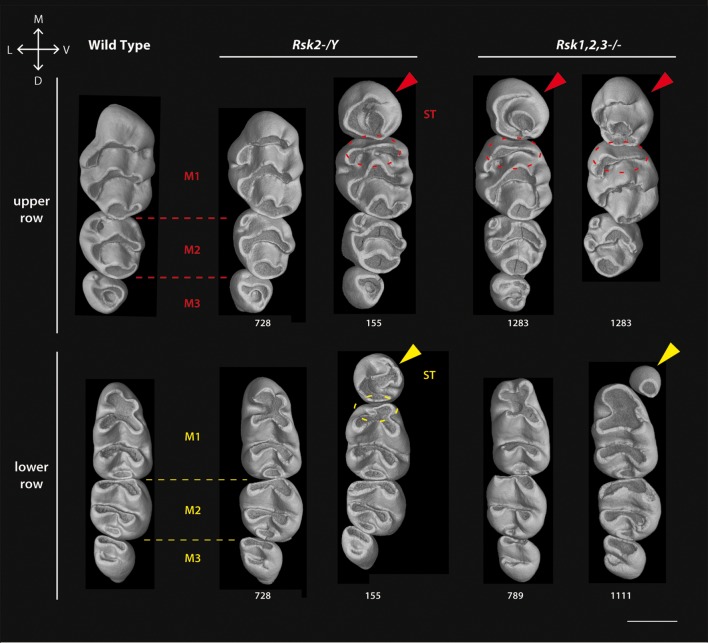
Variation of molar shape, size and number in *Rsk2-/Y* and *Rsk1,2,3-/-* mice analyzed by X-Ray microtomography. All molar rows are oriented in the same manner (top corresponds to mesial and left to lingual side). At the left are wild-type molars, other rows are mutants as indicated. Arrowheads point to the supernumerary teeth ST; dotted ellipses show the reduction of the mesial-most affected cusp. Scale bar: 0.7 µm.

We analyzed 15 *Rsk2-/Y* mice and 9 *Rsk1,2,3-/-* mutant mice (see Table 2 and Fig. 3). Supernumerary teeth (ST) occurred in all specimens except for two *Rsk2-/Y* mice (702, 731). ST were aligned with the molar tooth row and located just in front and at the contact of the first molars (M1). The phenotype penetrance was higher in the upper dentition (65% in *Rsk2-/Y* mice, 83% in *Rsk1,2,3-/-* mice) than in the lower dentition (31% and 11%, respectively). Shape and size of the supernumerary teeth were variable and ranged from a monocuspid tooth to a well-shaped molar-like tooth. ST were always smaller in size than the adjacent first molars, and they were sometimes, especially in the *Rsk1,2,3-/-* upper rows, as big as the M2. Inactivation of *Rsk2* alone was sufficient to generate the phenotype. When a ST was present, the mesial part of the first molar displayed altered crown morphology (Fig. 3). Even when the phenotype looked almost normal, a significant reduction in M1-M3 length was observed (Table 3). When ST occurred, the length mean value was always reduced for both M1 and M2 (Table 3). ST were always longer than M3. Molar width remained stable for M1, but was reduced for M2 and M3, except when only 3 molars were present in the mandible (Table 4). Shape abnormalities mainly occurred in mesial parts of the first lower (M1) and first upper (M1) molars in *Rsk2-/Y* mice. In upper M1, the central cusp of the mesial crest tended to be reduced, and the crest itself was more flattened (see red dotted circles in Fig. 3). Accordingly the mesial crest of the lower M1 also had an abnormal shape, because this crest tended to be strongly reduced when a ST occurred (see yellow dotted circles in Fig. 3). In triple mutants without ST (e.g. 789), the lower M1 displayed a mesial crest with vestibular cusp bigger than in WT.

**Table 2:** Presence (+) of a supernumerary tooth (mesial to the first molar) in *Rsk* mutant mice.

Mouse	Genotype	Upper Right	Upper Left	Lower Right	Lower Left
11	*Rsk2-/Y*	+ (4)	+ (4)	+ (4)	N
25	*Rsk2-/Y*	+ (4)	+ (4)	N	+ (4)
37	*Rsk2-/Y*	+ (4)	+ (4)	+ (4)	N
39	*Rsk2-/Y*	+ (4)	+ (4)	N	N
46	*Rsk2-/Y*	+ (4)	N	N	N
61	*Rsk2-/Y*	+ (4)	+ (4)	N	N
62	*Rsk2-/Y*	N	+ (4)	N	N
150	*Rsk2-/Y*	+ (4)	+ (4)	+ (4)	+ (4)
155	*Rsk2-/Y*	+ (4)	+ (4)	+ (4)	+ (4)
184	*Rsk2-/Y*	+ (4)	+ (4)	+ (4)	+ (4)
185	*Rsk2-/Y*	+ (4)	+ (4)	N	N
700	*Rsk2-/Y*	N	N	N	+ (4)
702	*Rsk2-/Y*	N	N	N	N
728	*Rsk2-/Y*	+ (4)	N	N	N
731	*Rsk2-/Y*	N	N	N	N
789	*Rsk1,2,3-/-*	+ (4)	+ (4)	N	N
793	*Rsk1,2,3-/-*	+ (4)	N	N	N
860	*Rsk1,2,3-/-*	+ (4)	N	+ (4)	N
883	*Rsk1,2,3-/-*	+ (4)	+ (3)	N	N
1105	*Rsk1,2,3-/-*	+ (4)	N	N	N
1111	*Rsk1,2,3-/-*	+ (3)	+ (3)	+ (4)	N
1283	*Rsk1,2,3-/-*	+ (3)	+ (4)	N	N
1139	*Rsk1,2,3-/-*	+ (3)	+ (4)	N	N
1159	*Rsk1,2,3-/-*	+ (4)	+ (3)	N	N

N: normal pattern of 3 molars M1, M2, M3. (3) means the presence of only 3 molars but the size of the second one is bigger (most probably there is a supernumerary tooth ST in front of M1, and M3 is missing).

**Table 3:** Comparison of each molar length, of the total molar field length, and the total length of M1+ M2, between wild-type (WT) mice and *RSk2-/Y* mutant mice with 3 or 4 molars.

	Mandibular molars WT	Mandibular molars Mutant 3M	Mandibular molars Mutant 4M	Maxillary molars WT	Maxillary molars Mutant 3M	Maxillary molars Mutant 4M
Mean ST			0.60			0.74
STD			0.01			0.02
Mean M1	1.44	1.37	1.14	1.56	1.38	1.14
STD	0.03	0.11	0.05	0.07	0.10	0.09
p-value		0.23	0.0004		0.01	0.001
Mean M2	0.89	0.85	0.79	0.92	0.85	0.82
STD	0.03	0.02	0.03	0.02	0.04	0.05
p-value		0.003	0.004		0.02	0.01
Mean M3	0.59	0.53	0.49	0.60	0.63	0.63
STD	0.02	0.03	0.04	0.03	0.03	0.05
p-value		0.0002	0.0004		0.96	0.15
Total	2.93	2.75	2.88	3.08	2.85	3.31
STD	0.04	0.15	0.28	0.10	0.13	0.06
p-value		0.0008	0.36		0.01	0.001
M1+ M2	2.33	2.10	2.15	2.48	2.30	1.96
STD	0.04	0.16	0.21	0.08	0.14	0.13
p-value		0.03	0.004		0.002	0.001

M1 size is reduced in mutants with 4 molars both in mandible and maxilla, but only in the maxilla of mutants showing 3 molars. M2 is reduced both in maxilla and mandible whatever the molar mutant phenotype. M3 mesiodistal dimension is reduced only in the mandible, irrespective of the presence of 3 or 4 molars. The total size of the molar field is reduced in the mandible and maxilla in mutant with 3 molars. The total size of the molar field is significantly increased in the maxilla in mutants with 4 molars. The total size of M1+M2 was always reduced when compared to the wild type value. All distances are in millimeters. Significant p-values are shown in bold.

M1 : molar 1; M2 : molar 2; M3 : molar 3; ST : supplementary tooth in front of M1; STD : standard deviation; Mutant 3M : mutant with 3 molars; Mutant 4M : mutant with 4 molars; Total : total length of all molars in the molar field.

**Table 4:** Comparison of each molar width (vestibulolingual dimension) between wild-type and *Rsk2-/Y* mutant mice with 3 or 4 molars.

	Mandibular molars WT	Mandibular molars Mutants 3M	Mandibular molars Mutants 4M	Maxillary molars WT	Maxillary molars Mutants 3M	Maxillary molars Mutants 4M
Mean ST			0.59			0.76
STD			0.09			0.04
Mean M1	0.79	0.77	0.82	1.04	1.01	1.06
STD	0.02	0.04	0.03	0.05	0.05	0.02
p-value		0.19	0.12		0.19	0.56
Mean M2	0.85	0.85	0.81	0.90	0.86	0.86
STD	0.02	0.02	0.03	0.02	0.05	0.03
p-value		0.89	**0.02**		**0.03**	**0.02**
Mean M3	0.60	0.57	0.58	0.61	0.58	0.54
STD	0.02	0.02	0.05	0.03	0.02	0.04
p-value		**0.006**	0.06		**0.02**	**0.01**

In mutants the width of M1 was not modified. The width of the maxillary M2 and M3 was reduced. In the mandible the width of M2 was lower only when 4 molars were present, and the width of M3 was lower only in mutants with 3 molars (all distances in millimeters; significant p-values in bold).

M1 : molar 1; M2 : molar 2; M3 : molar 3; ST : supplementary tooth in front of M1; STD : standard deviation; Mutant 3M : mutant with 3 molars; Mutant 4M : mutant with 4 molars.

The length of the molar row was significantly reduced in both the lower and upper jaws when no ST was present. When a ST occurred, the length of the molar row was almost normal in the mandible, but was significantly higher in the maxilla when compared to WT (Table 3). Some *Rsk1,2,3-/-* mouse specimens displayed 3 upper postcanine teeth, as in the WT, but the dental row encompassed maxillary ST-M1-M2, and lacked the M3. Consequently, the second tooth (M1) was bigger than neighbouring teeth ST and M2 (Fig. 3, specimen 1283).

#### Incisor phenotype

No statistically significant differences were revealed between right and left incisors in both groups (p>0.05). As a consequence, results from right and left incisors were merged (Table 5). No statistical difference was observed between WT and *Rsk2-/Y* (p>0.05). No obvious incisor anomaly was detected. Analysis of the incisor phenotype in *Rsk1,2,3-/-* mice was not performed due to a reduced number of available animals, visual inspection however did not revealed any incisor dental defects. The existence of incisor anomalies in R*sk1,2,3-/-* cannot be ruled out.

**Table 5:** Morphological analysis of mandibular incisors in WT and *Rsk2-/Y* mice.

		Li (mm)	hi (mm)	Hi (mm)	li (mm)	ti (mm)	lpa (mm^2^)
WT	Mean	11.33	1.11	2.96	9.38	0.65	10.36
	STD	2.34	0.07	0.20	1.56	0.02	1.07
*Rsk2-/Y*	Mean	11.29	1.14	2.97	9.05	0.65	10.18
	STD	1.66	0.05	0.21	1.65	0.03	1.33

No statistically significant differences were revealed between right and left incisors in both groups (p>0.05). As a consequence, results from right and left incisors were merged. No statistical difference was observed between WT and *Rsk2-/Y* (p>0.05).

(Li) length of the longest and external bow, (hi) height of median part of the incisor, (Hi) total height of the incisor, (li) horizontal length of the incisor joining the tip to the distal extremity of the root, (lpa) projected area in lateral view of the incisor, (ti) thickness the median part of the incisor. For more explanations on the measured distances, see Figure S3. STD : standard deviation.

#### Abnormal molar root development in *Rsk2-/Y* mice

Both the number and shape of molar roots also reflect the tooth identity. In WT mice the mandibular molars M1 and M2 have 2 roots, whereas M3 has only one root. The maxillary molars M1 and M2 have 3 roots. Variations in the root number of M3 occur both within and among various WT strains. The upper M3 molar usually has 2 or 3 roots, although sometimes it has only 1 root. Thus, the total number of molar roots is 5 per hemi-mandible, whereas it can vary from 7 to 9 per hemi-maxillary. In *Rsk2-/Y* mice, hemi-mandibles harbouring a ST had a total root number of 6, with the ST always having a single root. In the maxillary quadrant, the total root number varied from 8 to 9 when a ST was present. ST could have 1 or 2 roots, M1 had 2 roots, but the M2 root number remained stable with 3 roots (Table S1 in File S1). The presence of maxillary M1 with only 2 roots probably correlates with the occurrence of ST, often associated to a compression and a reduction of the mesial part of M1.

#### *Rsk2-/Y* and the diastema

The maxillary diastema was significantly reduced in *Rsk2-/Y* mice harbouring ST (Table 6). This was not the case for the mandibulary diastema, whatever the number of teeth.

**Table 6:** Upper and lower diastema size in wild-type and *RSk2-/Y* mutant mice with 3 or 4 molars.

	Mean size (mm)	STD	p-value
Mandibular diastema Wild-type	2.99	0.09	
Mandibular diastema Mutants with 3 molars	2.83	0.21	0.22
Mandibular diastema Mutants with 4 molars	2.71	0.1	0.26
Maxillary diastema Wild-type	5.45	0.18	
Maxillary diastema Mutants with 3 molars	5.33	0.21	0.49
**Maxillary diastema Mutants with 4 molars**	**5.01**	**0.21**	**0.0019**

Only the upper (maxillary) diastema from mutants with 4 molars was significantly smaller compared to wild-type (bold). STD : standard deviation.

### Expression patterns of *Rsk* genes during mouse odontogenesis

**Figure 4:**
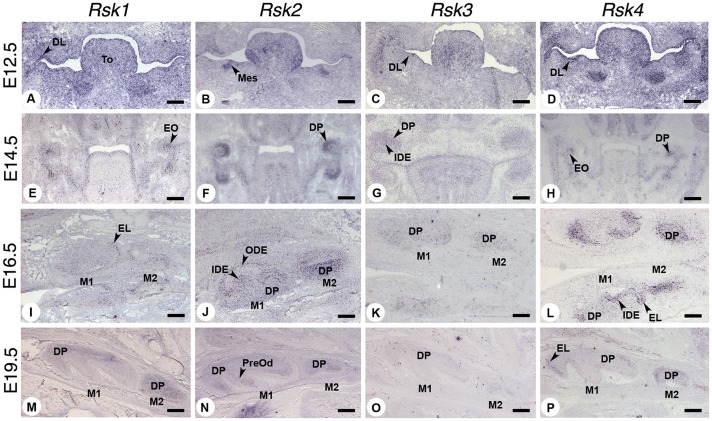
Expression patterns of *Rsk* genes during mouse molar odontogenesis at E12.5 (dental lamina), E14.5 (cap), E16.5 (bell) and E19.5 (late bell) stages, analyzed by *in situ* hybridization. Abbreviations: DL dental lamina, DP dental papilla, EL epithelial loop, EO enamel organ, IDE inner dental epithelium, M1 first molar, M2 second molar, Mes ectomesenchyme, ODE outer dental epithelium, PreOd preodontoblasts, To tongue. Scale bars 140 µm (A,B,C,D,I,J,K and L) and 250 µm (E,F,G,H,M,N,O and P).

**Figure 5:**
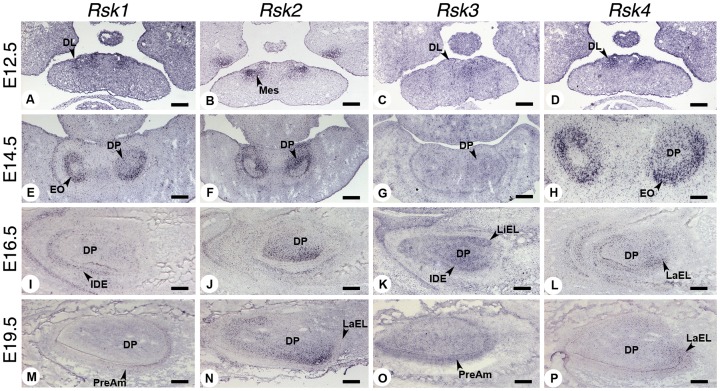
Expression patterns of *Rsk* genes during incisor odontogenesis at E12.5 (dental lamina), E14.5 (cap), E16.5 (bell) and E19.5 (late bell) stages analyzed by *in situ* hybridization. Abbreviations: DL dental lamina, DP dental papilla, EL epithelial loop, EO enamel organ, IDE inner dental epithelium, LaEL labial epithelial loop, LiEL lingual epithelial loop, Mes ectomesenchyme, PreAm preameloblasts. Scale bars 140 µm (A,B and C), 200 µm (D,E, F,G,I, J, K and L), 250 µm (M,N,O and P) and 50 µm (H).

To investigate whether the dental anomalies observed in the *Rsk*-deficient mice may correlate with distinct patterns of expression of *Rsk* genes during odontogenesis, we performed an *in situ* hybridization analysis of *Rsk1*, *Rsk2*, *Rsk3* and *Rsk4* mRNA transcripts at various stages of tooth development. All four genes were found to be expressed in specific areas of the tooth anlagen, as illustrated in Fig. 4 for the developing first molars, and Fig. 5 for the mandibular incisors. Their expression features are summarized in Table 7.

**Table 7:** *Rsks* expression patterns during odontogenesis.

	*Rsk1*				*Rsk2*				*Rsk3*				*Rsk4*			
	Inc	M1	M2	M3	Inc	M1	M2	M3	Inc	M1	M2	M3	Inc	M1	M2	M3
**E12.5**																
Oral epithelium	+	+											+	**+**		
Dental lamina	+	+							+ faint	+			+	**+**		
Mesenchyme					+	+				+			+			
**E14.5**																
Enamel organ	+	+											+	**+**		
IDE	+					+				+			+			
Enamel knot																
Dental Papilla	+ π faint				+	+			+	+			+	**+**		
Dental sac					+											
**E16.5**																
ODE	+					+	+									
IDE	+	+	+				+		+				+	**+**	**+**	
Epithelial loop	+	+	+						+				+	**+**	**+**	
Dental Papilla	+ lab				+ lab	+	+		+ π	+	+		+	**+**	**+**	
**E19.5**																
ODE																
SR																
SI																
IDE									+				+	**+**	**+**	
PreAm	+								+							
Am																
Epithelial loop	+	+							+				+ lab	**+**	**+**	
Od																
PreOd						+										
Dental papilla	+	+	+		+ lab	+	+		+	+	+		+ lab	**+**	**+**	
Dental sac					+											

+ : positive signal; lab: labial; π posterior area

IDE: inner dental epithelium; ODE: outer dental epithelium; SR: stellate reticulum; SI: stratum intermedium; PreAm: preameloblasts; Am: ameloblasts; Od: odontoblasts, PreOd: Preodontoblats

*Rsk2* transcripts were mainly localized in the mesenchymal compartment or dental papilla from E12.5 (dental lamina stage) to E19.5 (late bell stage). At E16.5 (bell stage), the transcripts were scattered in the area facing the epithelial loops or at the base of the cusps; the second molar dental mesenchyme was uniformly labelled. In the incisors the transcripts were also located in the mesenchyme, and the signal was more intense in the labial area and posterior area.

The expression of *Rsk1* and *Rsk4* was mainly epithelial, marking the inner dental epithelium and especially the epithelial loop areas. A faint signal was also observed for *Rsk4* in the posterior mesenchymal area facing the epithelial loops especially the labial loop for incisors (E16.5 and 19.5).

*Rsk3* transcripts were mainly visible in the dental papilla at E14.5 (cap stage), E16.5 (bell stage) and E19.5 (Figs. 4, 5 and Table 7). At E19.5 the transcripts were concentrated in the cervical part of the papilla for the first molar and had a wider distribution in the second molar mesenchyme.

### *Rsk2-/Y* supernumerary tooth is associated with an extra mesial placode

**Figure 6:**
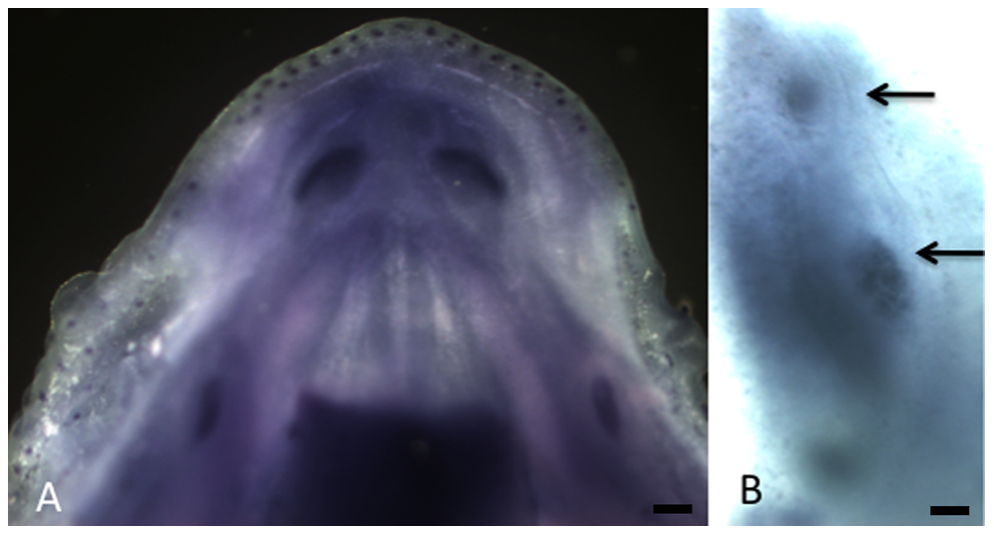
Whole mount in situ hybridization showing the molar and incisor placodes within the mandible at E14.5 in a wild-type mouse (A) and *Rsk2-/Y* mouse (B: detail of the right-side mandible) labelled with a *Shh* riboprobe. Two molar placodes are clearly visible in B (arrows) Scale bars 200 µm (A), 100 µm (B).

An extra tooth placode, and subsequent enamel knot labelled with *Sonic hedgehog* (*Shh*) as a marker, was clearly visible at E14.5, mesially to M1 in *Rsk2-/Y* mice when compared to WT littermates (Fig. 6). The presence of this extra placode was seen in nearly 30% of the analysed embryo and was not always simultaneously present on right and left sides of the jaw. During WT development the three molars (M1, M2 and M3) are forming from a unique molar placode. The presence of an additional dental placode mesially to the M1 placode likely corresponds to the primordium of ST developing in *Rsk2-/Y* mutants.

### Rsk2-/Y transcriptome analysis

**Figure 7:**
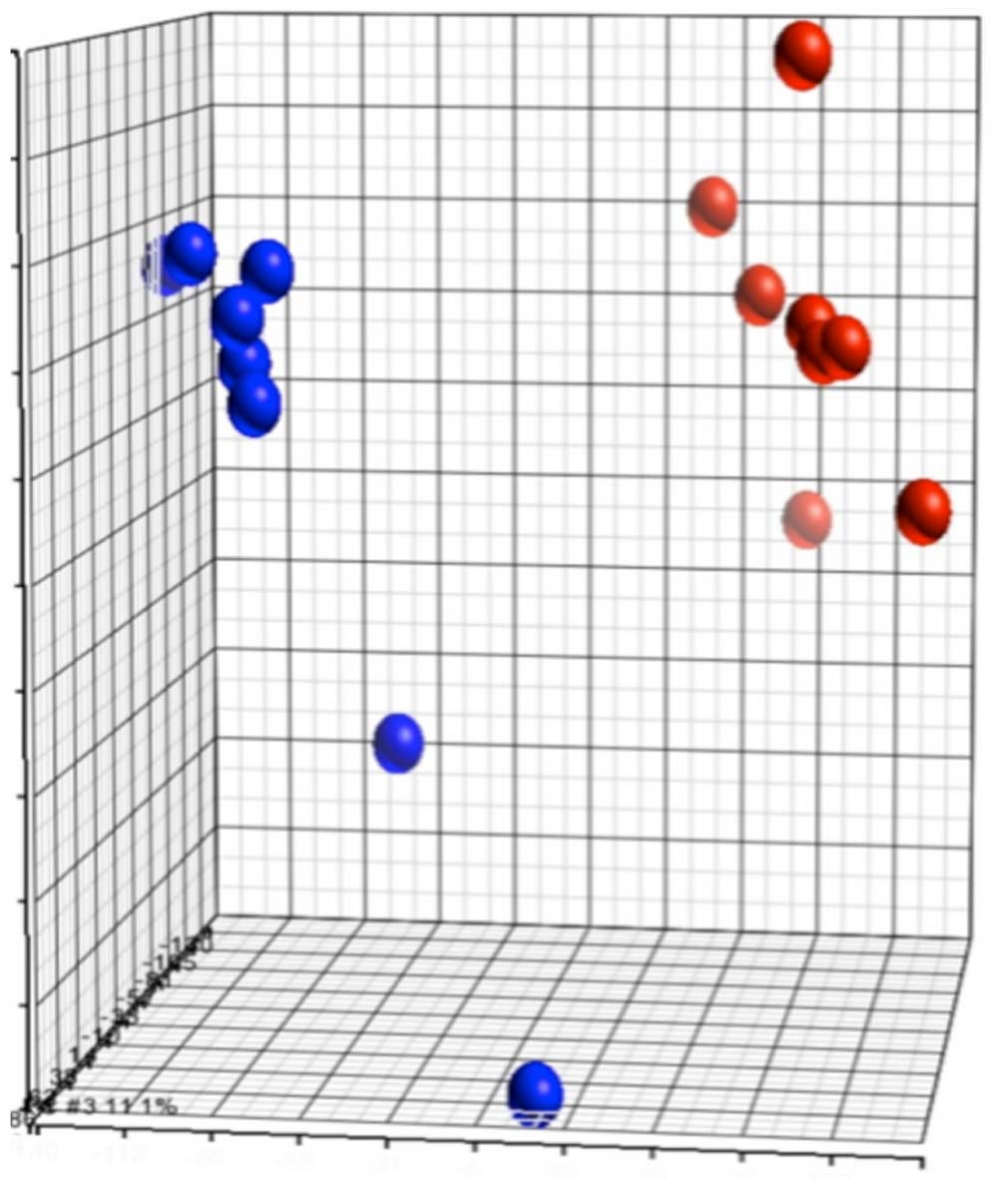
Principal component analysis (PCA) of transcriptomic data of wild-type versus *Rsk2-/Y* mandibular molars samples. Wild-type samples are represented as red balls, whereas mutant samples are represented in blue (X, Y and Z units are arbitrary units created by the software). The units are data-dependent and are generated by the software, which gives coordinates to each sample according to three axes that relate to the weight (inertia) of the decomposition into 3 principal components. For this analysis, samples segregate in two distinct groups, showing relevant transcriptional differences between WT and *Rsk2-/Y* samples.

We performed a comparative transcriptomic analysis (Affymetrix GeneChips) of wild-type and *Rsk2-/Y* mice, analyzing RNA isolated from dissected E14.5 mandibular molar tooth germs. By using a principal component analysis (PCA), it was possible to discriminate between the molar samples according to their genotype (wild-type versus *Rsk2-/Y*) (Fig. 7). 504 genes were retrieved as being differentially expressed, with fold changes exceeding ±1.2 and a false discovery rate below 0.1. The fold changes were however rather low, ranking between +2.044 and −1.98, suggesting that rather minimal molecular changes, at the transcriptome level, were taking place. These changes could be also minimized by a heterogeneous sampling for *Rsk2-/Y* molars, with putative phenotypes ranking as previously described between normality and a 3 molars with a supernumerary tooth phenotype; this phenotype was not known at the E14.5 cap stage, when molar germs were pooled for transcriptomic analysis. The most affected (“top ten”) genes, and a selection of additional genes being up- or down-regulated, are listed in Table 8 and Table 9.

**Table 8:** Overview of top ten genes differentially expressed in *Rsk2-/Y* and wild-type developing mandibular molars.

Top ten up-regulated genes	Fold change	P-value	Name
*Rdh1*	2.04458	5.22E-11	retinol dehydrogenase 1 (all trans)
*Dip2c*	1.82248	4.50E-11	DIP2 disco-interacting protein 2 homolog C (Drosophila)
*1700001J11Rik*	1.6761	2.92E-08	-
*Olfr319*	1.66441	1.56E-08	olfactory receptor 319
*Myh1*	1.64957	1.91E-06	myosin. heavy polypeptide 1 skeletal muscle
*Acaa1b*	1.62775	1.64E-07	acetyl-Coenzyme A acyltransferase 1B
*6430550D23Rik*	1.62394	3.80E-08	-
*Hmgb1*	1.61691	4.11E-10	high mobility group box 1
*4930555K19Rik*	1.58002	2.16E-09	-
*9630041A04Rik*	1.57948	3.38E-09	-
*BC022713*	1.57732	2.80E-07	cDNA sequence
*Gm9968*	1.56689	4.24E-12	predicted gene 9968
*Gm6718*	1.56677	4.74E-07	predicted gene 6718
*Naip2*	1.56543	3.72E-09	NLR family. apoptosis inhibitory protein 2

Top ten down-regulated genes	Fold change	P-value	Name
*Glycam1*	−1.55694	1.83E-10	glycosylation dependent cell adhesion molecule 1
*Sfrp5*	−1.5577	2.67E-06	secreted frizzled-related sequence protein 5
*Olfr459*	−1.56115	8.07E-10	olfactory receptor 459
*Fgl2*	−1.59412	7.32E-08	fibrinogen-like protein 2
*Ttc29*	−1.59842	6.35E-10	tetratricopeptide repeat domain 29
*2510003E04Rik*	−1.61995	2.51E-10	
*Tas2r117*	−1.63064	9.61E-12	taste receptor. type 2. member 117
*4930529M08Rik*	−1.65052	1.67E-07	
*4930529M08Rik*	−1.65555	3.89E-11	
*Rnf39*	−1.6586	7.87E-09	ring finger protein 39
*LOC100134990*	−1.67412	2.18E-09	selenoprotein K pseudogene
*Muc19*	−1.68802	5.59E-07	mucin 19
*Tpd52*	−1.74912	1.10E-10	tumor protein D52
*Sectm1b*	−1.9796	2.54E-06	secreted and transmembrane 1B

The table is highlighting genes showing the highest degree of enrichment (positive values) or down-regulation (negative values).

**Table 9:** Overview of other genes differentially expressed in *Rsk2-/Y* and wild-type developing mandibular molars.

Up-regulated genes	Fold change	P-value	Name
*Mdm2*	1.42143	6.43E-07	p53 E3 ubiquitin protein ligase homolog oncoprotein
*Neurog3*	1.39695	4.46E-08	neurogenin 3
*Pou6f2*	1.38695	2.35E-07	POU domain class 6 transcription factor 2
*Dffa*	1.38653	4.93E-10	DNA fragmentation factor alpha subunit
*Bdkrb1*	1.38509	4.15E-08	bradykinin receptor beta 1
*Ofd1*	1.37103	4.33E-08	oral-facial-digital syndrome 1 gene homolog; ciliogenic protein
*Zfp759*	1.35236	5.23E-07	zinc finger protein 759
*Cflar*	1.32394	7.32E-07	CASP8 and FADD-like apoptosis regulator
*Aak1*	1.23472	3.15E-06	AP2 associated kinase 1 positive regulation of Notch signaling pathway

Down-regulated genes	Fold change	P-value	Name
*Pde4a*	−1.20556	1.57E-07	phosphodiesterase 4A cAMP specific
*Eaf2/U19*	−1.23309	1.52E-06	ELL associated factor 2, ehrlich S-II transcriptional activator factor testosterone regulated apoptosis inducer and tumor suppressor
*Sp3*	−1.24825	9.34E-08	trans-acting transcription factor 3
*Ndufaf4*	−1.26049	5.41E-08	NADH dehydrogenase (ubiquinone) 1 alpha subcomplex. assembly factor 4
*Wnt11*	−1.28904	3.63E-08	wingless-related MMTV integration site 11
*Fgfbp3*	−1.30479	5.46E-07	fibroblast growth factor binding protein 3
*Ccna1*	−1.31914	9.33E-09	cyclin A1
*Trim69*	−1.35725	5.04E-08	tripartite motif-containing 69
*Npy5r*	−1.37768	5.33E-10	neuropeptide Y receptor Y5
*Zfp78*	−1.38882	1.95E-09	zinc finger protein 78
*Amhr2*	−1.46255	5.42E-09	anti-Mullerian hormone type 2 receptor transforming growth factor beta receptor signalling pathway
*L3mbtl4*	−1.48069	4.21E-08	l(3)mbt-like 4
*Zkscan17*	−1.48573	4.10E-08	zinc finger with KRAB and SCAN domains 17
*Ngf*	−1.49018	1.63E-08	nerve growth factor
*Nlrc5*	−1.53839	1.64E-08	NLR family. CARD domain containing 5 negative regulation of NF-kappaB transcription factor activity

The table is highlighting genes selected on the basis of their known involvement in pathways regulating odontogenesis.

The *Rdh1* gene encoding retinol dehydrogenase (RDH) 1 was found to be the most up-regulated gene in *Rsk2-/Y* mutant mice. In addition to retinol dehydrogenase activity, this enzyme has strong 3alpha-hydroxy steroid dehydrogenase- and weak 17beta-hydroxy steroid dehydrogenase- activities. *Rdh1* exhibits widespread and intense mRNA expression in many embryonic tissues including the neural tube, gut, and neural crest, as well as in adult mice [16]. Interestingly, retinoic acid (the end product of retinol oxidation initiated by RDH enzymes) is involved in tooth morphogenesis and differentiation, and abnormal retinoic acid levels can lead to dental anomalies [17], [18], [19], [20].

*Sectm1b*, encoding secreted and transmembrane protein 1B, was the most down-regulated gene in *Rsk2-/Y* mutant mice. This gene and protein had never been described previously as potentially involved in odontogenesis.

Among the differentially expressed genes selected (Table 9) as belonging to signalling pathways acting during tooth development (TGF-beta, FGF, Wnt, NF-kappaB) and or/cell cycle progression, *Eaf2* is a proto-oncogene acting together with Wnt4 in an autoregulatory feedback loop [21]. Inactivation of this gene causes numerous tumours in mice [22]. *Eaf2* was down-regulated in *Rsk2-/Y* mutants.

We tried unsuccessfully to confirm those results on a selection of genes by qRT-PCR, comparing three *Rsk2-/Y* and three WT molar RNA samples. This negative result might be explained by the heterogeneous nature of the *Rsk2-/Y* phenotype (as described above), the relative low level of expression changes, and may not invalidate the microarray data.

### *Rsk2* inactivation *in vitro*

In order to overcome the potential problem of *Rsk2-/Y* molar samples heterogeneity we decided to use an *in vitro* system, and inactivated *Rsk2* by microinjection and electroporation of shRNA in E14.5 molar tooth germs. *In vivo* electroporation in the mandible, as previously described by [23] was not suitable for our study, because of the lack of precision on the targeted zone and our aim to inactivate the gene directly within the tooth anlagen.

We optimized the experimental settings to inject and electroporate *Rsk2* shRNA in cap stage molars explanted and cultured *ex vivo*. Control explants were electroporated with a random shRNA construct. After electroporation the explants were placed into collagen drop culture to maintain their morphology [24] and kept in a defined culture medium for 24 h, before being processed for qRT-PCR analysis. Tooth morphology was preserved and neither apoptosis nor cell death were induced, as shown by activated caspase3 and TUNEL assays (data not shown).

**Figure 8:**
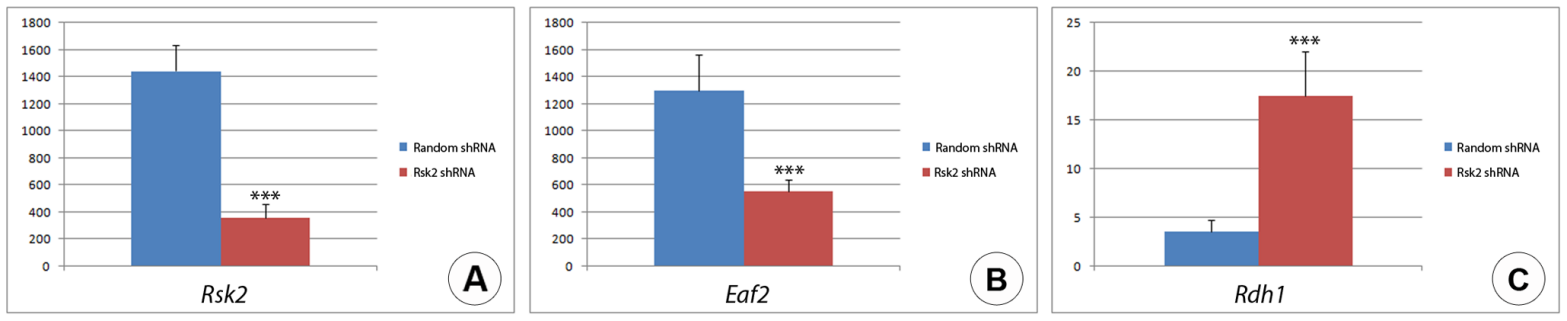
Summary of experiments involving *in vitro* inactivation of *Rsk2* and its consequences on target gene expression, as observed after microinjection in E14.5 mandibular molar explants of a *Rsk2* shRNA, followed by qRT-PCR analysis 24 h after injection. A: *Rsk2* was down regulated 4.01 fold after electroporation. B: *Eaf2* was significantly down-regulated, and C: *Rdh1* was up-regulated after electroporation. Histograms show expression levels in molars electroporated with the random shRNA (blue) and microinjected-electroporated explants with the *Rsk2* shRNA (red), with values normalized with respect to *Gapdh* expression. Data (mean ±SEM) were analyzed with Student t-test; ***p<0.001; **p<0.01; *p<0.05.

*Rsk2* shRNA electroporation efficiently diminished *Rsk2* expression by 75% (Fig. 8A). We then analyzed *Eaf2* and *Rdh1* expression, and found that *Eaf2* expression was down-regulated (75% decrease) (Fig. 8B), while *Rdh1* expression was up-regulated (50% increase)(Fig. 8C). These results were in accordance with the microarray data. The new combined technique allowed us not only to optimize a protocol for studying the molecular mechanisms of early tooth development, but also to set up a new approach for microarray validation.

## Discussion

### A peculiar dental phenotype

Mice are commonly studied mammals for investigating the mechanisms of tooth development and evolution. The orodental phenotype of mouse models of human genetic disorders also often mimics dental anomalies encountered in human rare diseases [14]. Basal placental mammals had a dental formula including three incisors, one canine, four premolars, and three molars per dentition quadrant [13], [25]. Through evolution, almost all mammals reduced their dental formulas, but lost functional teeth sometimes remaining as rudiments that begin to develop and then abort. The dentition of the mouse comprises 4 functional teeth in each quadrant (1 incisor and 3 molars separated by a toothless space called diastema). Rudimentary tooth primordia have been reported to develop in the distal part of the diastema through the initial stages of odontogenesis of WT mice. However their development ceases before the cap stage and they regress by apoptosis [26], [27], [28]. The tooth phenotype encountered in the *Rsk2-/Y* mutant mouse is remarkable by the occurrence of supernumerary teeth at the positions of the fourth premolars. Fourth premolars are still present in certain rodent groups such as squirrels, and have been lost through muroid rodent evolution [13].

### Rsk2 phenotype is similar to the tabby phenotype

Supernumerary teeth within the diastema were described in other mouse mutants for *ectodin, Lrp4, Sprouty2/4, Wise, Polaris*, and *Gas1* [29], [30], [31], [32], [33], [34], [35], [36]. Dysfunctions of the FGF, Shh, Wnt, and/or NF-kappaB signalling pathways are thought to induce formation of these supernumerary teeth within the diastema, indicating that the mouse maintains genetic potentialities that could be stimulated and induce the formation of supernumerary teeth [37], [38].

Ectopic teeth in the diastema are also observed in mice misexpressing *Eda* (ectodysplasin A; also known as Ta; Ed1; EDA1; XLHED; tabby) and *EdaA* receptor (Edar) [39], [40], [41], [42]. The dental phenotype of the *Rsk2-/Y* mouse resembles that encountered in the *tabby* mouse (a model for X-linked hypohidrotic ectodermal dysplasia- an ectodermal dysplasia characterized by an absence or dystrophic development of hair, nail, sweat- lacrymal- mammary- glands and tooth; OMIM #305100). Mutations in the human X-linked gene *ED1* (the *Eda* orthologue) are responsible for this disorder [43], [44], [45], [46]. Ontogenetic and phylogenetic data together suggest that the occurrence of functional supernumerary teeth in *tabby* mice diastema is caused by the persistence of the premolar rudiments that usually regress in WT mice. It has thus been proposed that this phenotype may represent an evolutionary throwback or atavism [43], [44], [45], [46].

Recently is has been shown that the number of teeth is regulated by fine-tuning of the level of receptor-tyrosine kinase signalling [32]. Known cytosolic RSK substrates include I-kappaB alpha [7], [47], [48], a member of the NF-kappaB pathway. This pathway is involved in development and in some human disorders including ectodermal dysplasias that affect ectodermal derivatives like the teeth, hair, salivary, mammary glands and skin. I-kappaB alpha has specifically been associated with an autosomal dominant form of anhidrotic ectodermal dysplasia and with T cell immunodeficiency [49]. The fact that I-kappaB alpha can be phosphorylated by RSKs, leading to increase of NF-kappaB activity, may therefore explain some of the dental anomalies encountered in the *Rsk2-/Y* transgenic mice and in Coffin-Lowry syndrome patients. However, the downstream signalling pathway(s) of Eda/Edar involved in tooth formation in the diastema are not yet known.

### The phenotype in the light of Coffin-Lowry syndrome

The mouse model for Coffin-Lowry syndrome, obtained by inactivation of the *Rsk2* gene and used in this study, was previously described by [11]. Mutant mice weigh 10% less and are 14% shorter than their wild-type littermates. This is in agreement with a role of RSK2 in organismal growth. These mice exhibit impaired learning and poor coordination, providing evidence that RSK2 plays similar roles at least for cognitive functions, in mouse and human. No previous description of the craniofacial or dental phenotype was so far reported. The craniofacial features described in Coffin-Lowry syndrome include a thick calvarium, hyperplastic supra-orbital ridges, hypertelorism, a flat malar region, and a prominent mandible. Clearly it is difficult in the light of our present observations, to relate the *Rsk*-knockout craniofacial phenotype to the anomalies encountered in patients. The small size of the skull could be associated to the general growth defect. However it is interesting to notice that in Coffin-Lowry patients, teeth agenesis and/or hypodontia is described. An apparent opposite phenotype between human patients and a mouse model has already been described in another rare disease, the cleido-cranial dysplasia syndrome, which is characterized by skeletal defects, numerous supernumerary teeth, and delayed tooth eruption. The mouse model of this disease (*Cbfa1*-KO) exhibits arrested tooth development [15]. Mostly loss of function mutations in *RSK2* both in human and mouse are responsible for the phenotype. Despite striking similarities mouse and human tooth development, cross-species comparison of mouse/human dentitions show they are clearly not alike. This suggests differential output and involvement of identical or superimposed pathways. These differences may account for this apparently opposite in nature dental phenotype i.e. hypodontia in human versus supernumerary teeth in mouse.

### Cell cycle progression and cell proliferation

It has been shown that tooth number, size, and shape are linked to cell proliferation and apoptosis events [50], [51], [52], [53]. It is interesting to note that *Rsks* genes are expressed throughout tooth development, especially in proliferating areas (this study, Figs. 4 and 5), either in the mesenchyme (*Rsk2, Rsk3*) or in the epithelial loops (*Rsk1, Rsk4*). The expression patterns described for the *Rsk* genes in other embryonic regions [12], [54], [55] also indicate a correlation with proliferative areas. *Rsk1* is highly expressed in tissues harbouring highly proliferating cells like liver, lung, thymus, and olfactory and respiratory epithelia. Particularly intense *Rsk1* expression is observed in the gut epithelium. *Rsk2* is expressed at very low levels throughout mouse development in dorsal root ganglia, thyroid gland, kidney, lung, liver and skeletal muscles. *Rsk3* is mainly expressed in the nervous system, but also in the thyroid gland and testis cords. RSK3 may regulate proliferation and seems linked to cellular differentiation of neuroepithelial cells during development [55]. *Rsk4* ubiquitous low expression was observed throughout development. In teeth *Rsk4* expression was rather specifically marking the epithelial loops.

It is well established that RSKs control cell proliferation through the regulation of mediators of the cell cycle [7]. RSK2 for example promotes cell cycle progression by phosphorylating c-Fos, a transcription factor regulating the expression of cyclin D1 during G1/S transition. RSK2 activates and phosphorylates p53 *in vitro* and *in vivo* and colocalizes with p53 in the nucleus. The RSK2-p53-histone H3 complex may likely contribute to chromatin remodelling and cell cycle regulation [56]. A list of phosphorylation substrates of the RSK isoforms was reported by [7] including c-Fos, NHE-1, Erα, p27^kip1^, Sos1, RanBP13, YB-1, Erp1, elF4B, rpS6, Bad, DAPK, Nur77, NFAT3, TIF-1A, ATF4, ATF1, MEF2c, Filamin A, Raptor, CCTβ, CRHSP24, Shank. Interestingly, alterations in the expression of a number of genes involved in cell cycle control (*Ccna1, Hmgb1, Mdm2, Tpd52*) or in cell growth (*Ngf, Bdkrb1, Amhr2*) is shown in our microarray analysis.

Increased proliferation and decreased apoptosis are necessary to develop supernumerary teeth, as seen by differential regulation of *Sprouty* genes [57]. The localisation of *Rsks* genes in proliferative areas, their role in the regulation of proliferation and apoptosis [58] as well as the interconnection of the involved pathways could explain that the deletion of *Rsk2* gene could induce the formation of supernumerary teeth and therefore reveal ancestral stages of murine dental evolution.

### RSK and the Ras/MAPK pathway

Altered ERK/MAPK signalling (with abnormally increased phosphorylation of ERK1/2) was recently reported in the hippocampus of the *Rsk2-/Y* mice [59]. RSK2 was also proven to exert a feedback inhibitory effect on the ERK1/2 pathway. Our microarray data did not indicate a similar trend at the level of the developing molar. *Ngf* was the only gene detected by our microarray analysis, known to be regulated at the transcriptional level by ERK1/2 and involved in tooth development [60].

### Unravelling target genes and involved pathways

The heterogeneity of the molar samples and the variability of the *Rsk2-/Y* molar phenotype has certainly interfered with the analysis of the microarray data and may have prevented the confirmation of target gene expression by qRT-PCR. However, genes involved in TGF-beta receptor signalling (Amhr2), or in the FGF (Fgfbp3) and Wnt (Sfrp5) pathways, were shown to be affected by the inactivation of *Rsk2*. These pathways are known to be involved in tooth development [61] and, when affected, are responsible for dental anomalies both in mouse models and human [62]. The *in vitro* assay we have developed, involving micro-injection and electroporation of a *Rsk2* shRNA in explanted molar tooth germs, proved to be a valuable system to assess quantitative changes in target gene expression.

## Conclusions

Analysis of *Rsk2-/Y* mutant mice revealed an important role of Rsk2 in craniofacial development, especially in dentition development and patterning. The loss of function of *Rsk2* allows the reappearance of supernumerary diastemal teeth considered as remnants of teeth lost over evolution. Our in situ hybridization analysis indicated that *Rsk2* expression pattern may correlate with proliferative areas of the developing teeth, consistent with a biological function of RSK2 in cell-cycle control and cell growth. Transcriptome profiling analysis was difficult because of the heterogeneity and variability of the *Rsk2-/Y* dental phenotype, however it provided candidate genes and pathways potentially involved in this phenotype. *In vitro* inactivation of *Rsk2* using shRNA was found to be a promising approach to address target genes and showed, in particular, an interference with an enzyme involved in the retinoid/steroid biosynthesis pathway. The fact that the regression of the fourth premolar seen in mouse is not found in all mammals, and is never observed in humans, may provide an explanation for the divergent phenotypes in *Rsk2-/Y* and human patients.

## Materials and Methods

### Patients and Ethics Statement

Patients attending the Reference centre for orodental manifestations of rare diseases, Pôle de médecine et chirurgie bucco-dentaire, Hôpitaux Universitaires de Strasbourg, or their legal representatives (parents, next of kin, caretakers or guardians) if minor and/or not in capacity to provide his or her own consent, give written informed consent for the use and publication of medical data. The picture proposed as figure 1 was uploaded in D4/phenodent database (CCTIRS positive report 11/09/2008, CNIL authorization 18/05/2009 (N°908416). A copy of the consent form can be downloaded at http://www.phenodent.org/consentement.php

### Animals and Ethics Statement

*Rsk2* gene targeting was previously described [11]. As in human, the mouse *Rsk2* gene is located on the X chromosome. Analyses were performed on *Rsk2-/Y* (“knockout”) male mice, with littermate wild-type (*Rsk2+/Y*) males used as control animals. Triple *Rsk1,2,3-/-* mutant mice were obtained by breeding of single knockout mice, themselves obtained by excision of exons three and four (*RSK1* knockout) or exon two (*RSK2* and *RSK3* knockouts) of the corresponding genes, leading to frameshift mutations [11]. The experiments were performed in accordance with the French national and European Laws and Directives Concerning Laboratory Animal Housing, Welfare and Experimentation and after approval from the CERBM-GIE: ICS/IGBMC Ethical Research Board.

### X-Ray microtomography

A cohort of 6 mutant *Rsk2-/Y* male mice (#150, 155, 700, 702, 728, 731) and 6 wild-type (WT) littermates (#149, 154, 699, 727, 729, 730), as well as 7 *Rsk1,2,3* compound mutant male mice (#789, 860, 883, 1105, 1111, 1139, 1283), were analyzed through X-ray micro-computed tomography (micro-CT) (GE eXplore Vision CT120 (General Electric, Waukesha, USA) for craniofacial exploration and Phoenix Nanotom (General Electric measurement and control, Billerica, USA) for tooth analysis). For craniofacial exploration the micro-CT was performed using 220 projections with an angle increment of 0.877 degrees (Parker mode) and one average frame per projection (70.0 kV, 32 mA, and exposition time of 16 milliseconds). 3D-reconstructions were done using a Feldkamp algorithm giving voxels of 100×100×100 µm^3^. For teeth a second micro-CT acquisition was performed with a smaller reconstructed voxel size (3×3×3 µm^3^) using 2 000 projections and three average frames per projection (100.0 kV, 70 µA, and exposure time of 500 ms). 3D-reconstructions were done using datos|x software and algorithm including beam hardening correction and ring artefact reduction.

Anatomical landmarks were identified as remarkable points (for instance cranial sutures) that could be easily recognized on each specimen. Fig. S1 (supplementary material) illustrates this step, showing on a 3D isosurface rendering the landmarks (red points) and their anatomical definitions. A similar approach was used for molar analysis (Fig. S2).

Calculation involved euclidean distances between selected pairs of points that could be compared to identify statistical significant differences between WT and mutant mice. The methodology is based upon previously described work [63], [64]. To avoid any bias in landmarks position, landmarks were placed two times by one investigator (intra-investigator reproducibility) and one time by another (inter-investigator reproducibility). As no statistical differences were observed between distances measured within or in between investigators, we decided to base our study on the average of the three measured distances. Statistical analysis comparing WT and mutant mice was performed using Mann-Whitney non parametric test. P-values lower than 0.05 were considered as significant.

### Analysis of incisor morphology

All image processing tasks were done using MicroView (GE Healthcare, Waukesha, USA). Right and left mandibular incisors were isolated by manual segmentation from mandible micro-CT acquisitions of the two mouse groups (6 *Rsk2-/Y* and 6 WT mice). Several measurements were achieved for morphology description, on the lateral and dorsal views of a 3D isosurface rendering of incisors (threshold value  = 700 Hounsfield units): the length of the longest and external bow (Li), the height of median part of the incisor (hi), the total height (Hi), the horizontal length joining the tip of the incisor and the distal extremity of the root (li), projected area in lateral view of the incisor (lpa) and the thickness of the median part of the incisor (ti) (Fig. S3). A Wilcoxon-Mann Whitney test was used to compare both groups.

### *In situ* hybridization on cryosections

I*n situ* hybridizations were performed according to an automated procedure described in [65], using cDNA plasmids described in Table S2 in File S1. Mouse embryos/fetuses (C57BL6) were collected at E12.5, E14.5, E16.5, and at the day of birth (referred to as E19.5). For E14.5 and older samples, the whole head was embedded in OCT 4583 medium (Tissue-TEK, Sakura) and frozen on the surface of dry ice. E12.5 embryos were fixed overnight in 4% paraformaldehyde (pH 7.5, w/v) in phosphate-buffered saline (PBS), cryo-protected by overnight incubation in 20% sucrose (w/v) in PBS, and cryo-embedded as described above. Cryosections (Leica CM3050S cryostat) at 10 µm were collected on Superfrost plus slides and stored at −80°C until hybridization. E12.5 and E14.5 samples were sectioned in a frontal plane, whereas other stages were sectioned sagitally.

### Whole mount in situ hybridization

Whole mount in situ hybridization of mandibles collected at E14.5 was performed with a digoxigenin-labeled *Shh* riboprobe as described [66], using an Intavis InSituPro robot (for details, see http://www.empress.har.mrc.ac.uk/browser/, Gene Expression section). The *Shh* template plasmid was kindly provided by A. McMahon (Harvard University).

### DNA microarray analysis

#### Tissue preparation

Embryos at E14.5 were dissected and mandibular molars were isolated. The tail was used for embryo genotyping. Individual tissue samples were frozen in liquid nitrogen and kept at -80°C until genotyping results were available and further used.

#### Microarray hybridization

To obtain enough RNA for hybridization on DNA microarrays, total RNA was extracted from molar tooth germs dissected manually (pooling samples from two independent wild-type or *Rsk2-/Y* embryos). Total RNA was extracted with the RNAeasy micro Kit (Qiagen). RNA quality was verified by analysis on the 2100 Bioanalyzer (Agilent). All samples displayed a RNA Integrity Number greater than 9.8. Eight *Rsk2-/Y* and 8 WT samples were hybridized and compared.

Biotinylated single strand cDNA targets were prepared, starting from 300 ng of total RNA, using the Ambion WT Expression Kit (Cat #4411974) and the Affymetrix GeneChip WT Terminal Labeling Kit (Cat #900671), according to Affymetrix recommendations. Following fragmentation and end-labeling, 1.9 µg of cDNAs were hybridized for 16 h at 45°C on GeneChip Mouse Gene 1.0 ST arrays (Affymetrix) interrogating 28,853 genes represented by approximately 27 probes spread across the full length of the gene. The chips were washed and stained in the GeneChip Fluidics Station 450 (Affymetrix) and scanned with the GeneChip Scanner 3000 7G (Affymetrix). Finally, raw data (.CEL Intensity files) were extracted from the scanned images using the Affymetrix GeneChip Command Console (AGCC) version 3.1. See also [67].

The data discussed in this publication have been deposited in NCBI's Gene Expression Omnibus (GEO) [68] and are accessible through GEO Series accession number GSE51034. (http://www.ncbi.nlm.nih.gov/geo/query/acc.cgi?acc=GSE51034)

#### Microarray analysis

CEL files were further processed with the Partek software to obtain principal component analysis (PCA) and to select and consider only genes with a signal value above 5 (20^th^ percentile of all expression values) in at least one sample. Genes were considered as differentially expressed if the false discovery rate from Benjamini and Hochberg test was under 0.1 and if the fold change was greater than 1.2 or lower than -1.2.

### *In vitro* culture and shRNA inactivation

E14.5 tooth germs were dissected and cultured in chemically defined culture medium as previously described [19]. The tooth explant consisted of both the enamel organ (epithelial component) and the attached dental mesenchyme. A DNA solution containing 1 µg of *Rsk2* shRNA (SABiosciences SureSilencing shRNA Plasmid for Mouse Rps6kA3 clone ID1– linearized pGeneclip Neomycin vector, insert sequence TGATGATACTCCAGAGGAAT) or a random shRNA (insert sequence GGAATCTCATTCGATGCATAC), 0.5% sucrose, and Fast Green (Sigma; 1/10.000) was injected into the developing molars with a Femtojet Eppendorf device (t = 0.2 s, P = 61 hPa). Two platinum needle electrodes (0.1 mm tip, Sonidel) were inserted into the tissue lying in a cover round platinum flat electrode (Sonidel). DNA was then transferred into the cells using an ECM 830 Electroporation System (BTX Harvard Apparatus), applying 1 set of 5 pulses. Electroporation settings were set to 5 V for 50 ms, spaced by 50 ms intervals. As negative control, explants were electroporated with a random shRNA sequence.

After electroporation, the explants were embedded into 14 µl rat collagen drops (BD Biosciences) containing 0.2 ng laminin (BD Biosciences), 50 µl DMEM 10x concentrated, 5 µl HEPES (1 mM), 60 µl 7.5% NaHCO_3_ (adapted from [24]). The collagen drops were kept on ice to prevent them from polymerizing. The collagen was then polymerized at 37°C for 10–15 min before addition of the culture medium.

All explants were subsequently cultured for 24 hours in a DMEM-HAM F12 medium supplemented with Vitamin C (18 mg/ml); L-Glutamine (200 mM); Streptomycine (1000 U/ml) and 20% fetal calf serum for optimal tooth growth. Explants were then processed for RT-qPCR analysis.

### Real-time quantitative RT-PCR

RT-PCR assays were performed in duplicate, comparing 3 wild-type and 3 *Rsk2-/Y* RNA samples (4 teeth were used in each sample for validation of microarray data), or 6 molar samples electroporated with *Rsk2* shRNA and 6 control samples (for shRNA experiments). RNA extractions were performed as previously described (ref. [67]). Oligo-dT primed cDNAs were generated using the Superscript II kit (Invitrogen) according to the manufacturer's protocol. Quantitative real-time PCR was achieved using SybrGreen and LightCycler 480 (Roche). The sequences of primers used for the various tested genes are given in Table S3 in File S1. A probe set for detection of mouse *Gapdh* mRNA (encoded by a housekeeping gene) was used for normalization. For each sample the ratio between signals for the gene of interest and *Gapdh* was calculated to normalize concentration values. To verify if genes were differentially expressed between electroporated and control samples, the average of ratios were then compared.

### Statistical tests

Data, presented as means ± SEM, were compared using Student t-test (for 2 groups comparison). P-values below 0.05 were considered as significant.

## Supporting Information

Figure S1 **Description of the craniofacial anatomical landmarks used for X-Ray microtomographic analysis of** ***Rsk*** **mutant mice**.
(TIF)

Figure S2 **Molar landmarks used for the analysis of X-Ray tomography images.**
(TIF)

Figure S3 **Three-dimensional isosurface rendering of a right mandibular incisor** from a wild-type mouse, in lateral (A) and dorsal (B) views, depicting the distances measured for the morphometric analysis.
(TIF)

File S1 **Supporting Information file containing Tables S1–S3.** Table S1 in file S1. Molar root numbers in WT and *Rsk2-/Y* mice. UR: upper right. UL: upper left. LR: lower right. LL: lower left. M1: first molar. M2: second molar. M3: third molar. ST: supernumerary tooth. Table S2 in file S1. Template plasmids used for *in situ hybridization*. Table S3 in file S1. Sequences of primers used for quantitative RT-PCR experiments.
(DOCX)
