# Supplementary figures and images for: RSK2 Is a Modulator of Craniofacial Development
Source: PLoS One. 2014 Jan 8;9(1):e84343. doi: 10.1371/journal.pone.0084343 (PMC3885557; doi:10.1371/journal.pone.0084343)

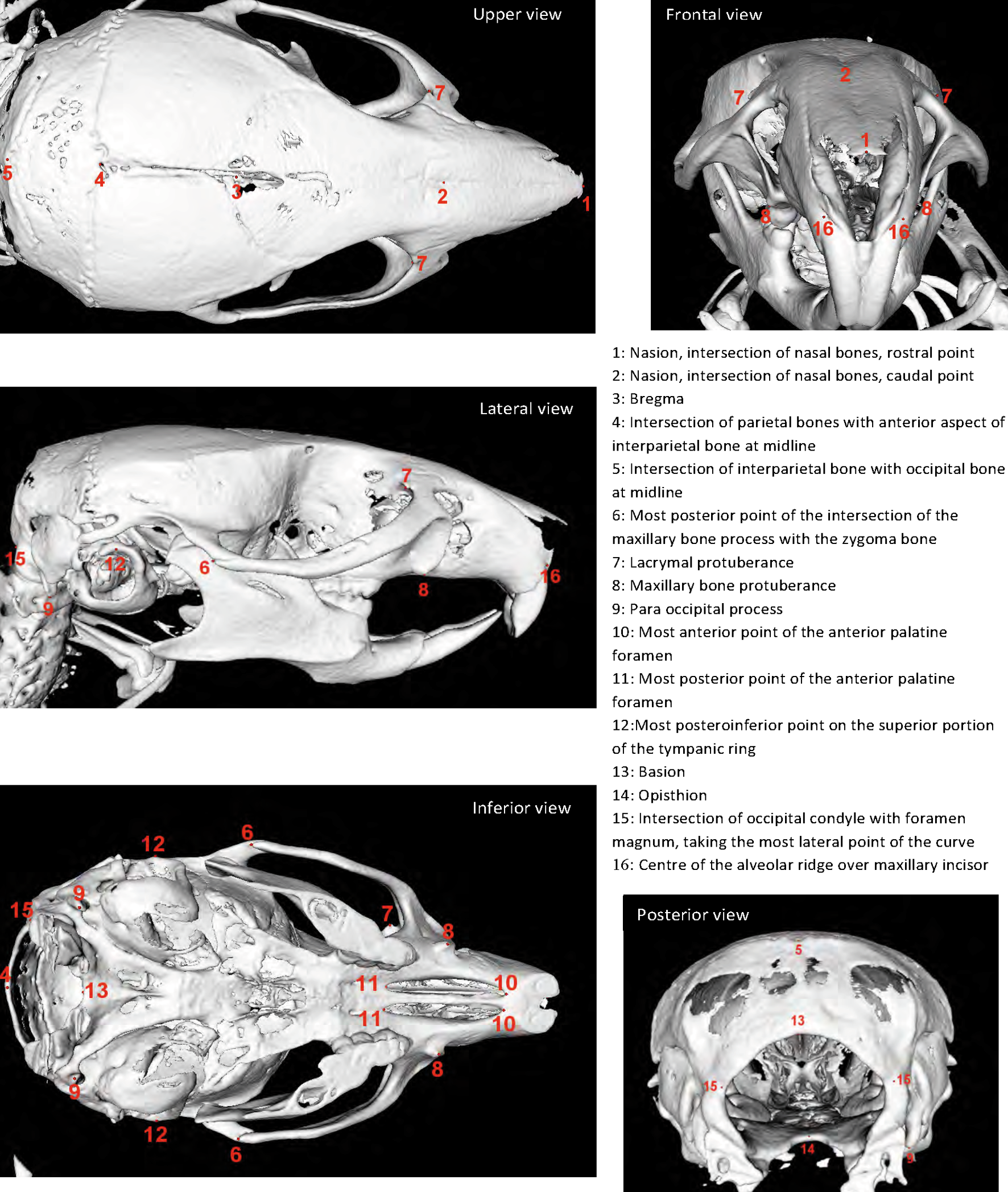

Supplement: Figure S1 — Description of the craniofacial anatomical landmarks used for X-Ray microtomographic analysis of Rsk mutant mice. (TIF) [file pone.0084343.s001.tif]

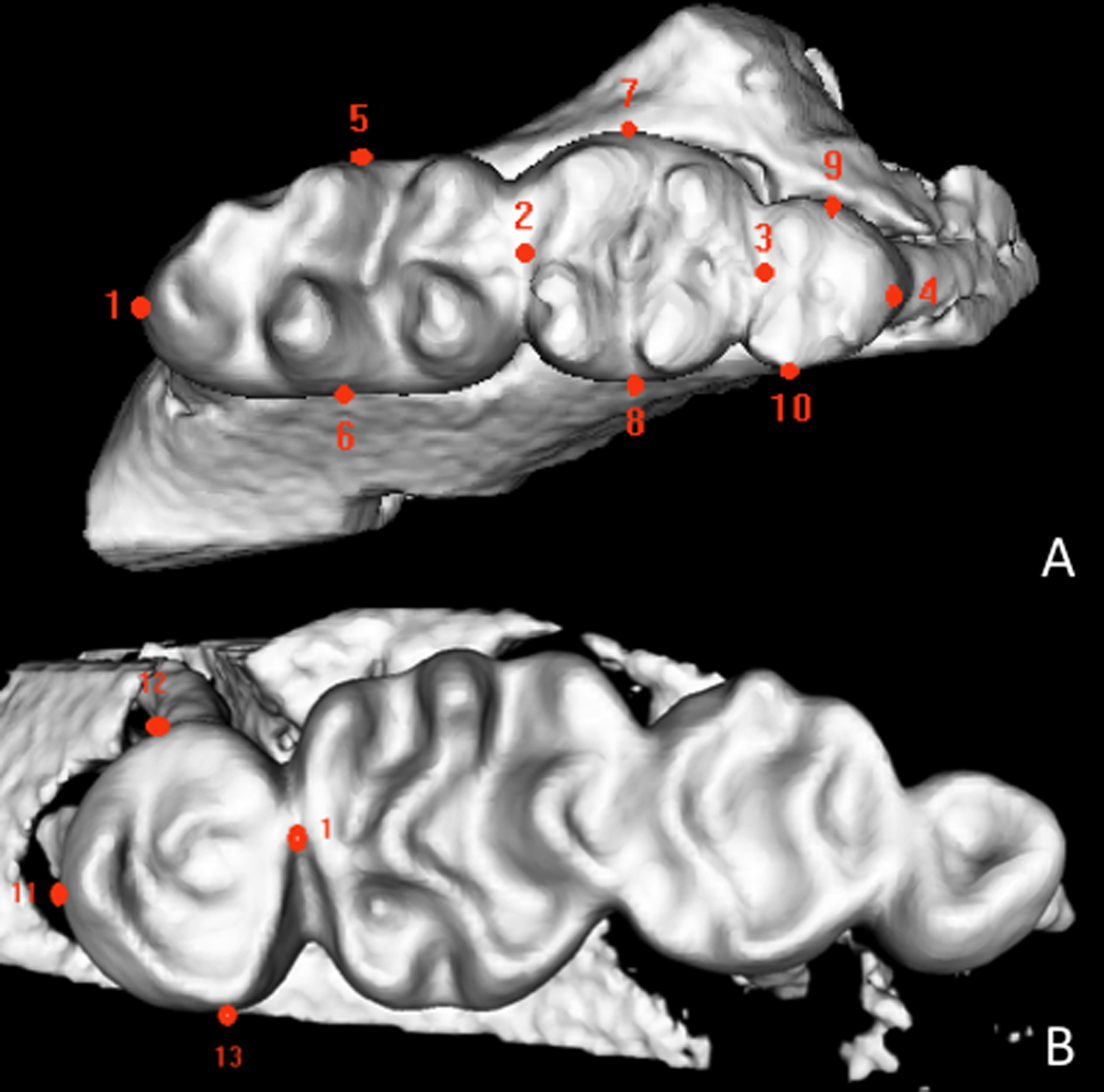

Supplement: Figure S2 — Molar landmarks used for the analysis of X-Ray tomography images. (TIF) [file pone.0084343.s002.tif]

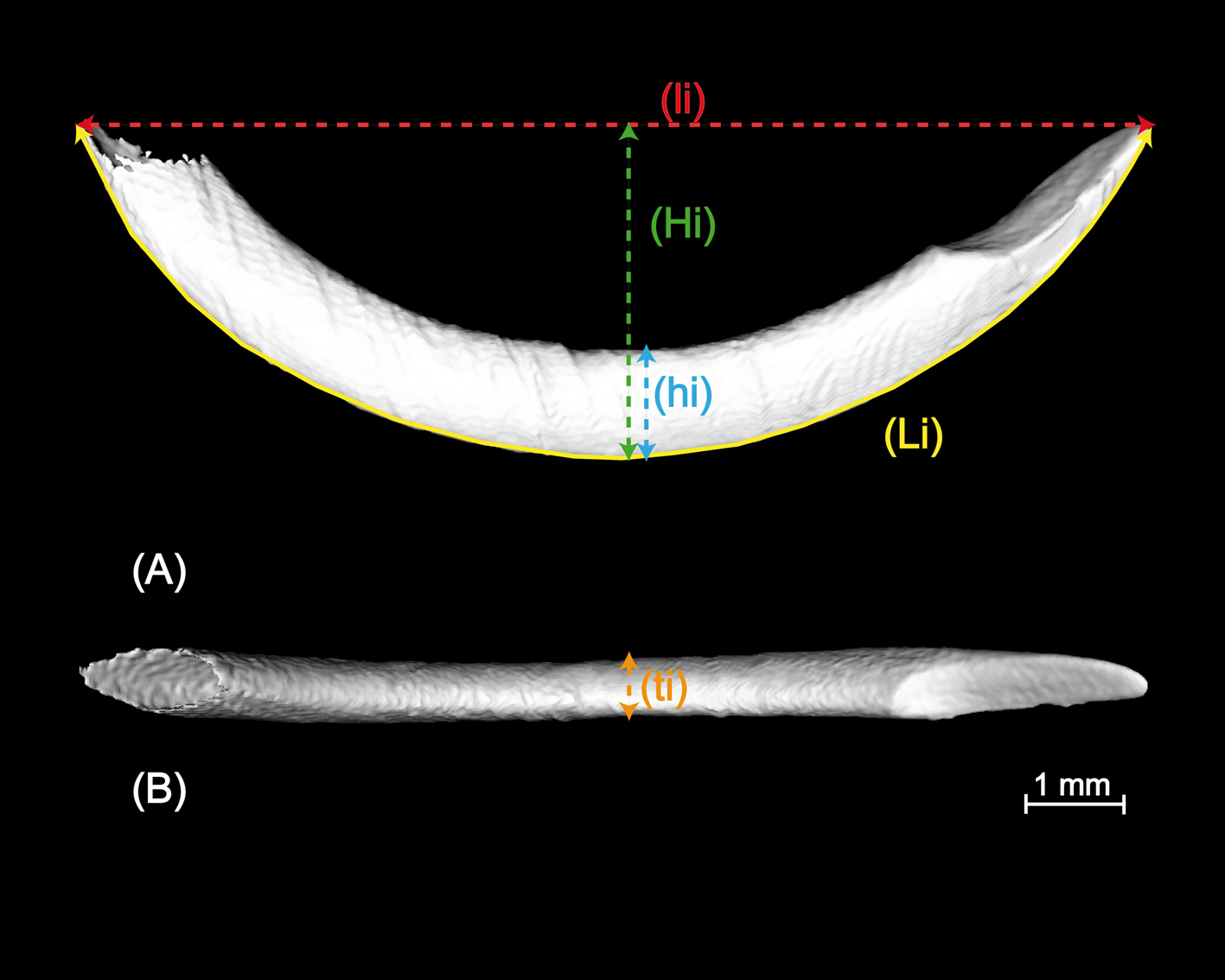

Supplement: Figure S3 — Three-dimensional isosurface rendering of a right mandibular incisor from a wild-type mouse, in lateral (A) and dorsal (B) views, depicting the distances measured for the morphometric analysis. (TIF) [file pone.0084343.s003.tif]
