# Supplementary material for: RSK2 Is a Modulator of Craniofacial Development
Source: PLoS One. 2014 Jan 8;9(1):e84343. doi: 10.1371/journal.pone.0084343 (PMC3885557; doi:10.1371/journal.pone.0084343)
Supplement: File S1 — Supporting Information file containing Tables S1–S3. Table S1 in file S1. Molar root numbers in WT and Rsk2-/Y mice. UR: upper right. UL: upper left. LR: lower right. LL: lower left. M1: first molar. M2: second molar. M3: third molar. ST: supernumerary tooth. Table S2 in file S1. Template plasmids used for in situ hybridization. Table S3 in file S1. Sequences of primers used for quantitative RT-PCR experiments. (DOCX) [file pone.0084343.s004.docx]

| WT # | M1 LR | M2 LR | M3 LR | Total LR | Total M1 + M2 | Total M1 and M2 + ST |
| --- | --- | --- | --- | --- | --- | --- |
| 149 | 2 | 2 | 1 | 5 | 4 |  |
| 730 | 2 | 2 | 1 | 5 | 4 |  |
| 729 | 2 | 2 | 1 | 5 | 4 |  |
| 727 | 2 | 2 | 1 | 5 | 4 |  |
| 699 | 2 | 2 | 1 | 5 | 4 |  |
| 154 | 2 | 2 | 1 | 5 | 4 |  |
| WT # | M1 LL | M2 LL | M3 LL | Total LL | Total M1 + M2 | Total M1 and M2 + ST |
| 149 | 2 | 2 | 2 | 6 | 4 |  |
| 730 | 2 | 2 | 1 | 5 | 4 |  |
| 729 | 2 | 2 | 1 | 5 | 4 |  |
| 727 | 2 | 2 | 1 | 5 | 4 |  |
| 699 | 2 | 2 | 1 | 5 | 4 |  |
| 154 | 2 | 2 | 1 | 5 | 4 |  |
| Mutant # | M1 LR | M2 LR | M3 LR | ST LR | Total LR | Total M1 and M2 + ST |
| 150 | 2 | 2 | 1 |  | 5 | 4 |
| 728 | 2 | 2 | 1 |  | 5 | 4 |
| 731 | 2 | 2 | 1 |  | 5 | 4 |
| 702 | 2 | 2 | 1 | 1 | 6 | 5 |
| 700 | 2 | 2 | 1 |  | 5 | 4 |
| 155 | 2 | 2 | 1 | 1 | 6 | 5 |
| Mutant # | M1 LL | M2 LL | M3 LL | ST LL | Total LL | Total M1 and M2 + ST |
| 150 | 2 | 2 | 1 | 1 | 6 | 5 |
| 728 | 2 | 2 | 1 |  | 5 | 4 |
| 731 | 2 | 2 | 1 | 1 | 6 | 5 |
| 702 | 2 | 2 | 1 |  | 5 | 4 |
| 700 | 2 | 2 | 1 |  | 5 | 4 |
| 155 | 2 | 2 | 1 | 1 | 6 | 5 |
| WT # | M1 UR | M2 UR | M3 UR | Total UR | Total M1 +M2 | Total M1 and M2 + ST |
| 149 | 3 | 3 | 3 | 9 | 6 |  |
| 730 | 3 | 3 | 2 | 8 | 6 |  |
| 729 | 3 | 3 | 2 | 8 | 6 |  |
| 727 | 3 | 3 | 2 | 8 | 6 |  |
| 699 | 3 | 3 | 2 | 8 | 6 |  |
| 154 | 3 | 3 | 3 | 9 | 6 |  |
| WT # | M1 UL | M2 UL | M3 UL | Total UL | Total M1 + M2 | Total M1 and M2 + ST |
| 149 | 3 | 3 | 3 | 9 | 6 |  |
| 730 | 3 | 3 | 2 | 8 | 6 |  |
| 729 | 3 | 3 | 2 | 8 | 6 |  |
| 727 | 3 | 3 | 2 | 8 | 6 |  |
| 699 | 3 | 3 | 1 | 7 | 6 |  |
| 154 | 3 | 3 | 2 | 8 | 6 |  |
| Mutant # | M1 UR | M2 UR | M3 UR | ST UR | Total UR | Total M1 and M2 + ST |
| 150 | 2 | 3 | 2 | 2 | 9 | 7 |
| 728 | 2 | 3 | 2 | 2 | 9 | 7 |
| 731 | 2 | 3 | 2 |  | 7 | 5 |
| 702 | 2 | 3 | 2 |  | 7 | 5 |
| 700 | 3 | 3 | 2 |  | 8 | 6 |
| 155 | 2 | 3 | 2 | 1 | 8 | 6 |
| Mutant # | M1 UL | M2 UL | M3 UL | ST UL | Total UL | Total M1 and M2 + ST |
| 150 | 2 | 3 | 2 | 1 | 8 | 6 |
| 728 | 2 | 3 | 2 |  | 7 | 5 |
| 731 | 3 | 3 | 3 |  | 9 | 6 |
| 702 | 3 | 3 | 3 |  | 9 | 6 |
| 700 | 3 | 3 | 2 |  | 8 | 6 |
| 155 | 2 | 3 | 2 | 2 | 9 | 7 |

**Table S1 : Molar root numbers in WT and *Rsk2-/Y* mice.** UR: upper right. UL: upper left. LR: lower right. LL: lower left. M1: first molar. M2: second molar. M3: third molar. ST: supernumerary tooth.

**Table S2 : Templates for *in situ* hybridization**

| GENE | Sequence | Vector | RNA polymerase |
| --- | --- | --- | --- |
| *Rsk1* | 3097-2282 | pCMV.SPORT6 | T7 |
| *Rsk2* | 3025-2117 | pT7T3D-Pacl | T3 |
| *Rsk3* | 5374-4403 | pCMV.SPORT6 | T7 |
| *Rsk4* | 4330-3405 | pCMV.SPORT6 | T7 |

**Table S3 : Primer sequences of genes analyzed by quantitative RT-PCR.**

| Primer | Sequence 5' to 3' |
| --- | --- |
| *RDH1 QF* | AACACGCAGAGCAATGAGGAG |
| *RDH1 QR* | TAGATGTGGCGAACCATGCC |
| *SP3 QF* | TCAAGTAGTCGCTAATGTGCCT |
| *SP3 QR* | GAACTTCCCGAGAGTCCCAAA |
| *POU6F2 QF* | GGACAGATTATTGGGACCATTCC |
| *POU6F2 QR* | GGTGTGATAGGCTGTACTTGAAG |
| *NLRC5 QF* | GTGCCAAACGTCCTTTTCAGA |
| *NLRC5 QR* | AGTGAGGAGTAAGCCATGCTC |
| *EAF2 QF* | GAGGCTGATGCTACTTGTCAC |
| *EAF2 QR* | CTCACTGTCGCTTTCTGACTC |
| *PDE4A QF* | ACATTTCCAACACGTTCCTAGAC |
| *PDE4A QR* | CCGGTGTGTACCAGCTTTTTC |
| *CFLAR QF* | GGTGGAAGAGTGTCTTGATGAAG |
| *CFLAR QR* | CCCTGACGTTAGGTGCAGC |
| *AAK1 QF* | GCAATGGGGTGAAATGTGCC |
| *AAK1 QR* | TGTGCCCTGATAGGTCTCTCA |
| *MDM2 QF* | TGAAGTTGTTAAAGTCCGTTGGA |
| *MDM2 QR* | CTGCTGCTTCTCGTCATATAACC |
